# A two‐dimensional multiwell cell culture method for the production of CYP3A4‐expressing hepatocyte‐like cells from HepaRG cells

**DOI:** 10.1002/prp2.652

**Published:** 2020-09-21

**Authors:** Keiko Ooeda, Musashi Kubiura‐Ichimaru, Saori Tsuji, Shota Okuyama, Mao Yamashita, Akari Mine, Fumihiko Kawamura, Takafumi Ueyama, Masako Tada

**Affiliations:** ^1^ Stem Cells & Reprogramming Laboratory Department of Biology Faculty of Science Toho University Funabashi Japan; ^2^ Reagents Business KAC Co. Amagasaki Japan

**Keywords:** a dual‐color reporter, *CYP3A4*, *CYP3A7*, cytochrome P450 enzymes, HepaRG, hepatocyte‐like cells, high‐content screening

## Abstract

Cytochrome P450 enzymes (CYP) function in drug metabolism in the liver. To evaluate numerous drug candidates, a high‐content screening (HCS) system with hepatocyte‐like cells (HLCs) that can replace adult human hepatocytes is required. Human hepatocellular carcinoma HepaRG is the only cell line capable of providing HLCs with high CYP3A4 expression comparable to that in adult hepatocytes after cell differentiation. The aim of this study was to design an ideal multiwell culture system for HLCs using transgenic HepaRG cells expressing the *EGFP* coding an enhanced green fluorescent protein under *CYP3A4* transcriptional regulation. HLCs were matured on five different types of 96‐well black plates. Culturing HLCs on glass‐bottom Optical CVG plates significantly promoted cell maturation and increased metabolic activity by twofold under two‐dimensional (2D) culture conditions, and these features were enhanced by 2% collagen coating. Three plates for three‐dimensional (3D) cell cultures with a gas‐exchangeable fabric or dimethylpolysiloxane membrane bottom formed multiple round colonies, whereas they were ineffective for *CYP3A4* expression. Under optimized conditions presented here, HLCs lost responsiveness to nuclear receptor‐mediated transcriptional induction of *CYP3A4*, suggesting that *CYP3A4* transcription has already been fully upregulated. Therefore, HepaRG‐derived HLCs will provide an alternative to human hepatocytes with high levels of CYP3A4 enzyme activity even under 2D culture conditions. This will improve a variety of drug screening methods.

**Abbreviations**
2D: two‐dimensional
3D: three‐dimensional
CYP: cytochrome P450 enzymes
DMSO: dimethylsulphoxide
HBs: hepatoblasts
HCS: high‐content screening
HLCs: hepatocyte‐like cells
iPSCs: induced pluripotent stem cells
LC‐MS/MS: liquid chromatography tandem‐mass spectrometry
PHHs: primary human hepatocytes
RIF: rifampicin

## 1 INTRODUCTION

Drug metabolism occurs mainly in the liver. Drugs undergo structural alterations catalyzed by the metabolic enzyme cytochrome P450 (CYP), which is expressed in the intestine and in hepatocytes. CYP3A4 metabolizes approximately 50% of marketed drugs.^1^, ^2^ Therefore, drug discovery and development require evaluation of the cytotoxicity and drug‐drug interactions of candidate compounds before and after absorption in the human body using human hepatocytes. A high‐content screening (HCS)‐based assay for many candidates is necessary to select nontoxic and effective compounds, as it would improve cost and time effectiveness in the early stages of drug discovery and development. HCS evaluates multiparameter images acquired with a motor‐driven microscope and extracts quantitative data. Therefore, HCS requires a large amount of adult primary human hepatocytes (PHHs) expressing CYP3A4. However, PHHs are limited in number because of their poor proliferative ability, and individual and lot differences negatively affect data reproducibility.^2^, ^3^ Therefore, it is indispensable to develop a model of adult PHHs that can be stably supplied, are scalable and qualitatively uniform and express CYP3A4. Moreover, certain chemicals can alter the susceptibility to a drug by promoting or inhibiting the pharmacological actions of coadministered drugs.^4^, ^5^, ^6^, ^7^, ^8^ To predict the clinical effect of drug‐drug interactions, it is also necessary to evaluate the possible transcription‐inducing function of *CYP3A4*.

HepaRG cells have characteristics similar to those of human fetal liver cells, hepatoblasts (HBs), and addition of 1‐2% dimethylsulphoxide (DMSO) to the culture medium induces their differentiation into adult‐type HLCs.^9^, ^10^ HepaRG HLCs are the only cultured cells expressing CYP3A4 at high levels similar to those expressed by adult PHHs.^11^, ^12^ In previous work by our group, we generated transgenic HepaRG cells, and determined the presence of HB‐like cells expressing fetal‐type *CYP3A7* and adult‐type HLCs expressing *CYP3A4* by detecting the expression of *DsRed* and *EGFP*, respectively, before and after cell differentiation.^13^, ^14^ The transgenic CYP3A4G/7R HepaRG provides an easily applicable and inexpensive system to determine the degree of cell differentiation according to the color shift from red fluorescence to green fluorescence.^14^, ^15^ Compounds that induce *CYP3A4* transcription can also be conveniently evaluated based on the fold increase of normalized green fluorescence intensity. When cell numbers seeded in each well are strictly controlled, and enough number of wells are analyzed for each condition, total green fluorescence intensity per area can represent relative *CYP3A4* transcriptional levels among wells. Moreover, cell death induced by hepatotoxic compounds can be better monitored by the reduction of total green fluorescence intensity per area, as both of transcription and cell numbers are reduced. In previous work by our group, we generated transgenic mice for the CYP3A7‐DsRed BAC reporter gene, which contains the complete transcriptional regulatory units of *CYP3A7* and *CYP3A4* and the gene body of *CYP3A7* was replaced with *DsRed*. Then we found that 5% carbon tetrachloride (CCl_4_) injection induces red fluorescence reactivation in the liver of adult transgenic mice *via* transient HB expansion during liver regeneration.^16^ Therefore, red fluorescence recovery could be used as a tool to identify hepatotoxic compounds. In both cases, achieving automatic scanning with a confocal microscope and limiting light leakage from adjacent wells require the use of multiwell plates with a flat and thin bottom and a black wall for HLC culture.

In recent years, multiwell plates suitable for 3D cell culture were developed and are commercially available. It is believed that these are necessary because hepatocytes that form microspheroids tend to exhibit high enzyme activities comparable to those of hepatocytes under biological conditions.^17^ Therefore, the combination of CYP3A4‐expressing HLCs, 3D culture of HLCs, and a multiwell plate with a flat surface is essential for reliable screening. However, an optimal system remains to be developed; in particular, a multiwell plate suitable for live‐cell imaging is lacking.

In this study, we aimed to establish a multiwell cell culture system for HepaRG‐derived HLCs showing high levels of CYP3A4 enzyme activity. We first prepared a sufficient amount of homogenously differentiated HepaRG‐derived HLCs. Specific numbers of HLCs were seeded in each well of five 96‐well plates and one 24‐well plate, to compare re‐colonization status and subsequent re‐maturation efficiency.

The relative expression levels of *CYP3A4* were evaluated by measuring total green fluorescence intensity and *EGFP* and *CYP3A4* mRNA expression levels using confocal microscopy and reverse transcription‐quantitative PCR (RT‐qPCR), respectively. The enzyme activity of CYP3A4 was assessed by measuring the amount of 1'‐hydroxymidazolam, a probe for measuring CYP3A4‐mediated metabolism using liquid chromatography tandem‐mass spectrometry (LC‐MS/MS). To identify the cell culture system capable of providing the most sensitive evaluation of *CYP3A4* transcription induction, induction rates were compared in HLCs cultured on six plates and treated for 48 hours with rifampicin (RIF).

## 2 EXPERIMENTAL METHODS

### 2.1 Cell culture

All media used for HepaRG cell culture were purchased from Biopredic International, Rennes, France. Cell culture media used in this study were 710, 720, and 640, and MIL502. The contents of these media are not disclosed by the manufacturer, but they differ in serum and DMSO concentration and are specified for cell proliferation, cell differentiation, transcription induction assays, and mass spectrometry.

Transgenic and wild‐type (WT) HepaRG cells at the passage (P)11 and P19 were used, respectively. Cells were seeded at 1.5 to two times the recommended number of cells to maintain differentiation potency. After 2‐week (2 W) cell culture in medium 710 at 37°C and 5% CO_2_, the DMSO concentration in the medium was gradually increased from 0.1% to 0.4% from 3 to 4 days before subculture. To produce HLCs, cells were seeded in fifteen 25‐cm^2^ cell culture flasks at a density of 0.5 × 10^6^ cells/cm^2^ and cultured in medium 710 for 2 W. The cells were then cultured in the 0.4% DMSO medium for 3 days, the 1% DMSO medium for 2 days, and finally in the 1.7% DMSO medium for 9 days. Therefore, the initial cell differentiation of HepaRG cells to HLCs was completed in 4 weeks (4 W).

Four‐week‐old CYP3A4G/7R and WT HepaRG HLCs were gently dissociated with 0.05% trypsin‐EDTA (1×) (Thermo Fisher Scientific). The dissociation of tight adhesion among HLCs induces cell dedifferentiation from HLCs to growing HB‐LCs, so mildly dissociated cell aggregates were transferred to multiwell plates. An aliquot of the 4‐W differentiated cell mixture collected from the 15 flasks was placed into each well of a multiwell plate (7.2 × 10^4^ cells/well). All plates used are commercially available as listed in Table 1 (Cosmo Bio or Thermo Fisher Scientific). Additional cell cultures on assay plates were achieved as described in the Results section. Immediately prior to use, some of plates were coated with 2‐20% Cellmatrix Type I‐A (Nitta Gelatin).

**Table 1:** Cell culture plates used for HepaRG‐derived hepatocyte‐like cell culture

	Plate	Symbol	Wells/plate	Well size (cm^2^)	Cells/well (×10^4^)
1	C‐lect™ stem cell passage	C	96	03	72
2	VECELL G plate	G	96	03	72
3	VECELL H plate	H	96	03	72
4	VECELL HAG plate	HAG	96	03	72
5	Optical CVG	O	96	03	72
6	Preset VECELL	V	24	15	288

### 2.2 Cell culture for the *CYP3A4* induction test

During the last 72 hours of culture, cells were cultured in medium 640 containing 10 µmol L^−1^ RIF or 0.1% DMSO. For LC‐MS/MS analyses, cells were incubated in medium MIL502 containing 50 μmol L^−1^ midazolam at 37°C during the last hour of culture. RIF and midazolam were dissolved in DMSO and stored at −20°C.

### 2.3 Fluorescence microscopy and image analysis

Fluorescence microscopic images were captured with an A1 confocal microscope (Nikon, Tokyo, Japan). The mean and standard deviation of the total fluorescence intensity of the area within each well was calculated from the images using Image J software, version 2.0.0‐rc‐69/1.52p (an open‐source program for image analysis provided by the National Institutes of Health, Bethesda).^18^

### 2.4 RNA extraction and RT‐qPCR analyses

Total RNA was isolated using the RNeasy Mini Kit (Qiagen, Venlo, Netherlands), and cDNA was prepared from 0.5 µg of total RNA using ReverTra Ace® qPCR RT Master Mix with gDNA Remover (TOYOBO). cDNA from 10 ng of RNA was amplified in 25 µl reactions using KOD SYBR® qPCR Master Mix (TOYOBO) and a 7500 Real‐Time PCR System (Applied Biosystems). *ACTB* was used as an internal control. Primer sets used for RT‐qPCR are listed in Table 2.

**Table 2:** PCR primers used for gene expression analyses

Set	Gene	Size	Primer name	Sequence (5' to 3')
1	*EGFP*	101 bp	GFP q‐RT F	GAAGCGCGATCACATGGT
			GFP q‐RT R	CCATGCCGAGAGTGATCC
2	*CYP3A4*	86 bp	hqCYP3A4‐3A7‐F	TTCATCCAATGGACTGCATAAAT
			hqCYP3A4‐R	TCCCAAGTATAACACTCTACACAGACAA
3	*ACTB*	115 bp	h/mActb‐F	CTTCTACAATGAGCTGCGTG
			h/mActb‐R	GAAGGTCTCAAACATGATCTGG

### 2.5 LC‐MS/MS analyses

The medium MIL502 supplemented with 50 μmol L^−1^ midazolam was prepared before use. An aliquot (0.5 × 10^6^) of the original 4‐W HLCs was incubated for 1 hours at 37°C in a 500 μl midazolam mixture. The remaining HLCs were cultured in 96‐ or 24‐well plates. After cells were matured in multiwells, 125 µl of midazolam mixture per well was added in four wells of a 96‐well plate and 250 µl midazolam mixture per well was added in two wells of a 24‐well plate and incubated for 1 hour at 37°C. The culture supernatant was diluted with methanol at a ratio of 1:3, and cell debris was removed from the sample by centrifugation at 1700 *g* for 15 minutes at 4°C. The supernatant was diluted with water containing 3 μmol L^−1^ α‐hydroxymidazolam‐D4, an internal standard. Levels of 1'‐hydroxymidazolam and α‐hydroxymidazolam‐D4 were quantified using LC‐MS/MS. Liquid chromatography experiments were performed with a prominence UFLC system (SHIMAZU) coupled with QTRAP5500 (SCIEX). The detailed methods were described previously.^15^

### 2.6 Immunocytochemistry (ICC)

Immunocytochemistry was performed using WT HepaRG‐derived HLCs to exam protein expression, because transgenic cells express fluorescent. Cells were fixed with PBS containing 2% (w/v) paraformaldehyde (PFA) for 5 min and washed with PBS. Permeabilization was performed with PBS containing 0.1% Triton X for 10 min, followed by washing with PBS. After incubation in PBS containing 2% skim milk for 30 min, cells were incubated with rabbit polyclonal anti‐human CYP3A4 antibody (Enzo Life Sciences), mouse monoclonal anti‐human cytokeratin 19 antibody (CK19; DakoCytomation), mouse monoclonal anti‐human albumin (ALB; Sigma‐Aldrich), mouse monoclonal anti‐keratin K8/K18 (CK8/18; PROGEN), hepatocyte nuclear factor 4 alpha (HNF4A), and mouse monoclonal anti‐GFP (B2; Santa Cruz Biotechnology), diluted 1:500 in PBS with 2% skim milk for 1 hours at room temperature. Then, samples were incubated with the secondary antibodies Alexa Fluor® 488‐conjugated rabbit IgG and Alexa Fluor® 546‐conjugated mouse IgG (Invitrogen) diluted 1:500 in PBS with 2% skim milk for 1 hours at room temperature. Samples were washed three times with PBS containing 0.05% Tween‐20 for 10 minutes. DNA was stained with Hoechst 33 258 solution (Merck).

### 2.7 Statistics

Data are presented as the mean ± SD. The *Z* test Excel formula was used to evaluate the statistical significance of differences between two samples. Significance was determined using equal‐variance *Z* values on both sides. The correlation coefficient (*R*) was calculated using the software in Microsoft Excel. Values of *P* < .01 were considered significant. *P < .01; **P < .001.

## 3 RESULTS

### 3.1 Effect of culture plate type on colony morphology of HepaRG‐derived HLCs

**FIGURE 1:**
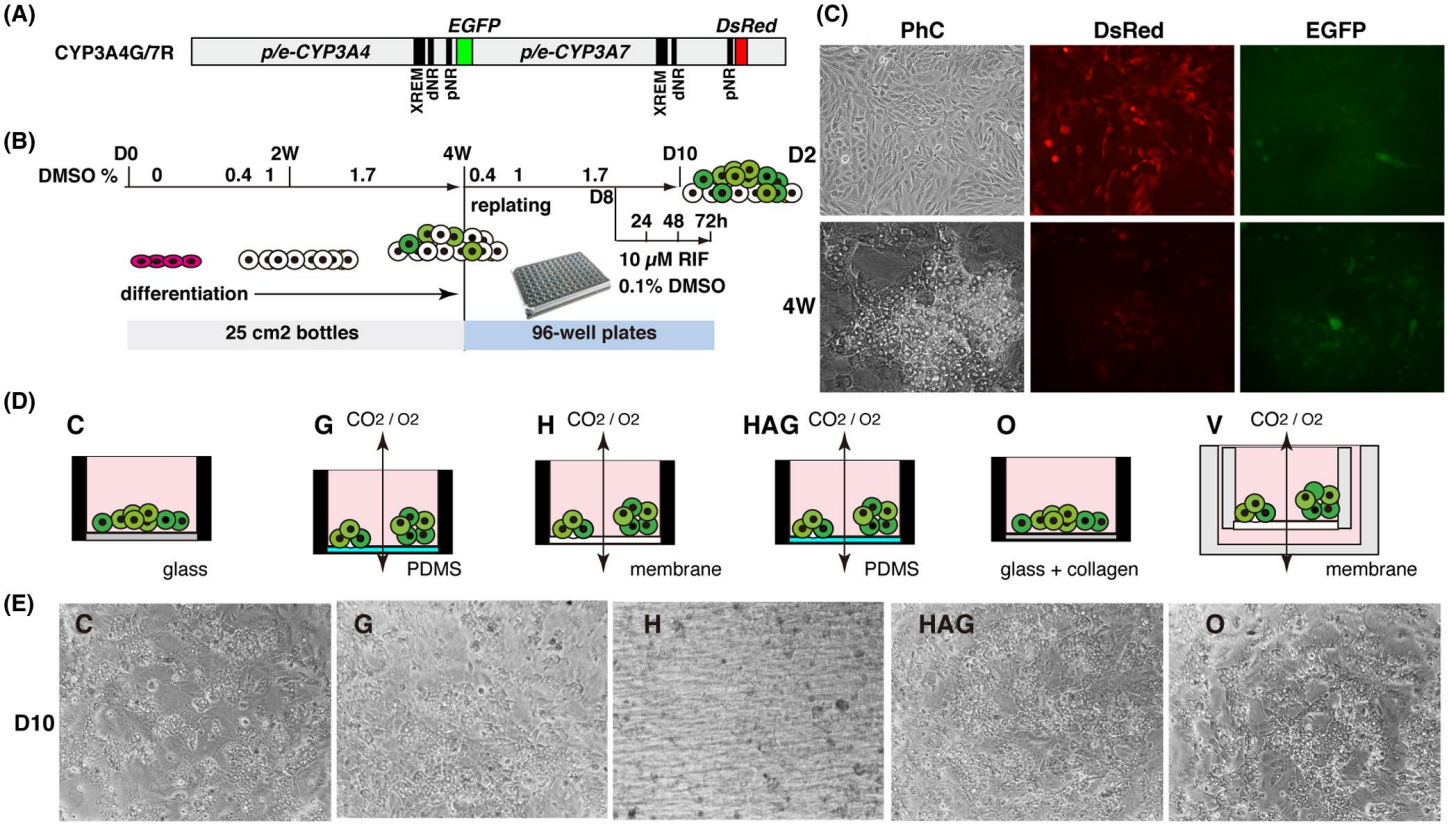
Effect of cell culture plate type on cell proliferation and colony formation of hepatocyte‐like cells (HLCs) differentiated from transgenic CYP3A4G/7R HepaRG. A, Structure of the CYP3A4/7R BAC reporter. *EGFP* and *DsRed* were used as transcriptional reporter genes for *CYP3A4* and *CYP3A7*, respectively. B, Schematic showing the cell culture schedule. C, Morphology and fluorescent images of differentiated CYP3A4G/7R HepaRG cells. D2, 2 days; 4 W, 4 weeks; PhC, phase contrast image. D, Illustrations showing the characteristics of a well in each plate. E, Colony morphology of D10 HLCs after replating

In this study, five 96‐well plates with different characteristics were used to compare the cell maturation abilities of *CYP3A4*‐expressing HLCs (Table 1). First, transgenic CYP3A4G/7R HepaRG cells were cultured in 15 culture flasks with a bottom area of 25 cm^2^ (Figure 1A,B). The 4‐W old cells showed loss of red fluorescence in standard cell culture flasks, although they were not clearly positive for green fluorescence (Figure 1C). The 4‐W cells were gently dissociated and collected from 15 culture flasks. Aliquots of uniformly mixed differentiated cells were then seeded at a density of 7.2 × 10^4^ cells/well of 96‐well plates. The medium 710 containing 0.4% DMSO was used during the first 2 days after seeding. Thereafter, to promote HLC maturation, cells were cultured in the 1% DMSO medium for 1 day and then cultured in the 1.7% DMSO medium by day 10 (D10).

Because the BAC reporter gene CYP3A4G/7R contains a complete transcriptional element, *EGFP* transcription can be enhanced by the *CYP3A4* transcriptional inducer RIF (Figure 1A). Therefore, we also attempted to select plates suitable for identifying compounds that enhance *CYP3A4* transcription *via* nuclear receptors. On D8, a medium containing 0.1% DMSO or 10 µmol L^−1^ RIF was added to three wells per plate (n = 3) and further cultured for 3 days under comparable conditions (Figure 1B). Since RIF is dissolved in DMSO, the culture medium with RIF contained 0.1% DMSO, and the 0.1% DMSO medium was used as the negative control in the CYP3A4 induction test. Cells were used for analyses at 24, 48, and 72 hours after RIF treatment.

Optical CVG (O) plates (Thermo Fisher Scientific) were pretreated with 10% Cellmatrix Type I‐A as a first trial. Collagen‐coated O plates and untreated C‐lect™ Stem Cell Passage (C) plates (Cyntellect®) were used as 2D culture plates. Both possess a thin glass bottom and black walls. For 3D culture, we used three plates purchased from VECELL®. The hybrid H (H) plate possesses a porous membrane at the bottom. The VECELL G (G) plate, which can promote the formation of numerous cell aggregates, has a gas‐permeable dimethylpolysiloxane (PDMS) membrane at the bottom. The HAG plate (HAG) also has a PDMS membrane bottom, which promotes cell growth more efficiently than the G plate (Figure 1D,E). The properties of the Preset VECELL® (V) plate were previously reported using hepatoma HepG2 cells.^19^ Cells were seeded in a 24‐well V plate at a comparable density to that in a 96‐well plate. Cell morphology and the fluorescence of HepaRG‐derived HLCs were undetectable under a microscope because, in V plates, cells are cultured in the scaffold of a polytetrafluoroethylene mesh coated with salmon collagen (Figure 1D,V). Colony morphology of D10 HLCs differed depending on the material of the bottom surface in each plate (Figure 1E). On the collagen‐coated O plate, remarkable cell proliferation of differentiated cells was observed immediately after re‐seeding. Subsequently, the cord‐like structure of HLCs was formed by D10 cells only on O plates.

### 3.2 Effect of plate type on maturation into HLCs

**FIGURE 2:**
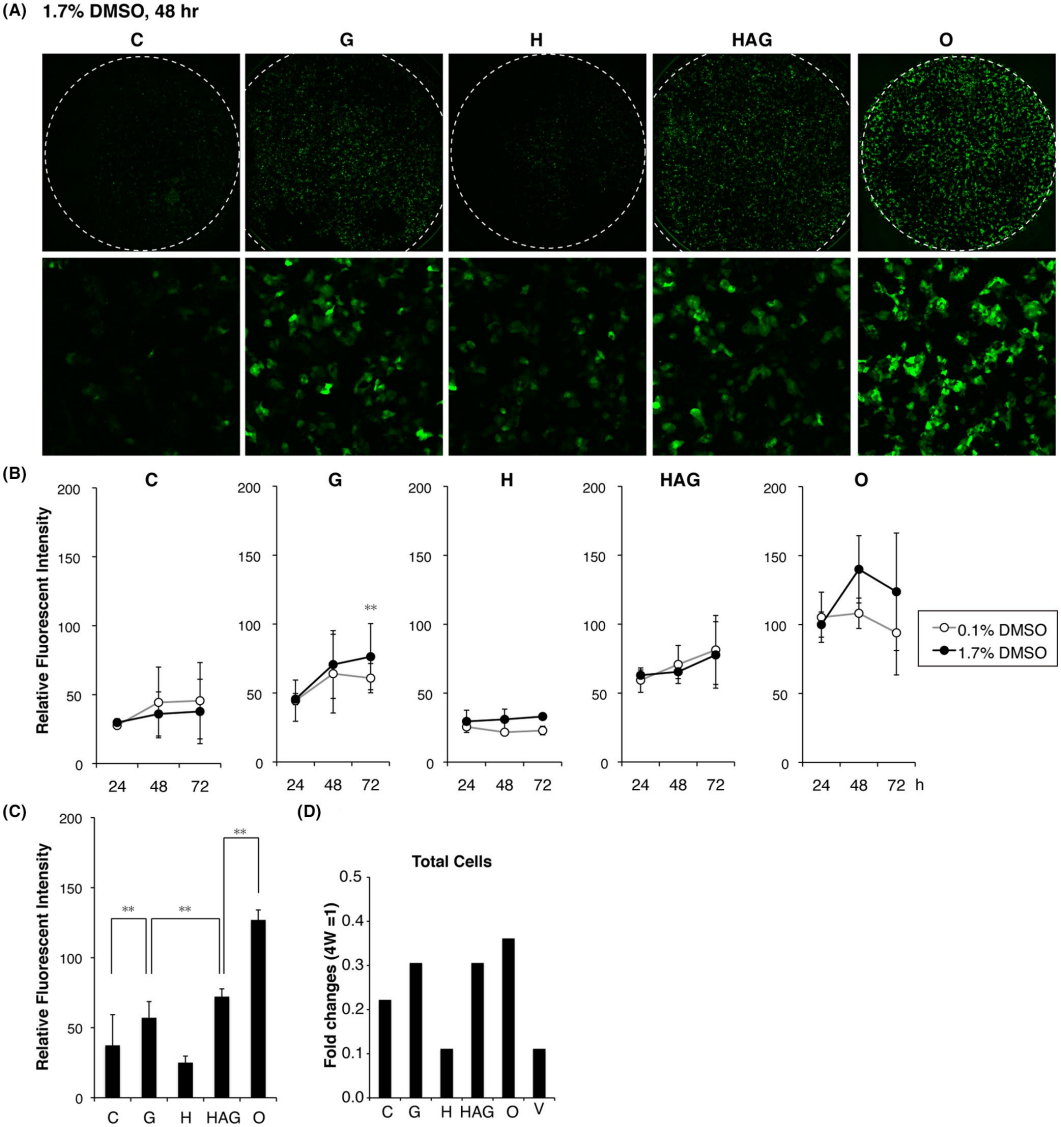
Effect of cell culture plate type on maturation into hepatocyte‐like cells (HLCs) expressing EGFP fluorescence. A, EGFP‐positive cell images in each well of 96‐well plates captured by confocal microscopy (upper). Enlarged view of the center of the well (bottom). B, Semi‐quantitative fluorometric evaluation of EGFP‐positive HLC frequencies. Relative values were calculated according to the average value obtained from D9 cells cultured on O plates in the 1.7% DMSO medium taken as 100 (n = 3). Black circles, cells were cultured in the 1.7% DMSO medium; open circles, DMSO concentration reduced from 1.7% to 0.1% on the last 3 days of the cell culture period. C, Comparison of relative fluorescence intensity of EGFP in D10 HLCs between five plates (n = 3). D, Relative cell numbers were estimated based on the fluorescence intensity of DNA staining with Hoechst 33 258

In CYP3A4G/7R HepaRG cells, *EGFP* expression is regulated by the *CYP3A4* promoter and enhancer, and green fluorescence therefore reflects the expression levels of *CYP3A4*. Images of D10 cells were acquired using a confocal microscope (Figure 2A). The number of EGFP‐expressing HLCs was overwhelmingly larger in an O plate than in a C plate. EGFP‐positive cells were detected to the same extent on two plates, G and HAG. Fewer EGFP‐positive cells appeared on an H plate. Since the bottom of H plates consists of an opaque and porous membrane, it is impossible to evaluate the fluorescence intensity on H plates accurately. The number of EGFP‐positive cells was minimal on C plates.

Next, to compare the relative amounts of EGFP‐positive HLCs between plates, the average fluorescence intensity values per well were calculated using Image J (n = 3). To analyze the effect of 1.7% DMSO on cell maturation, we compared the fluorescence levels between 1.7% DMSO‐treated wells and 0.1% DMSO‐treated wells at 24, 48, and 72 hours in D9, D10, and D11 cells, respectively. Each value was expressed relative to an average of values obtained in D9 HLCs cultured on an O plate in the 1.7% DMSO medium (O = 100). The results demonstrated that 1.7% DMSO is required for continuous cell maturation of HLCs on G and O plates, and maximizes EGFP‐positive HLCs cultured on O plates on D10 (Figure 2B, 48 hours). Comparison of total green fluorescence levels on D10 between plates showed that EGFP‐positive HLCs were twofold more abundant in HAG plates than in C plates, and approximately fourfold more abundant in O plates than in C plates (Figure 2C, *P* < .001). Cell growth that was estimated based on the fluorescence intensity of nuclear staining with Hoechst 33 258 varied between the plates. The cells grew well on O plates (Figure 2D).

**FIGURE 3:**
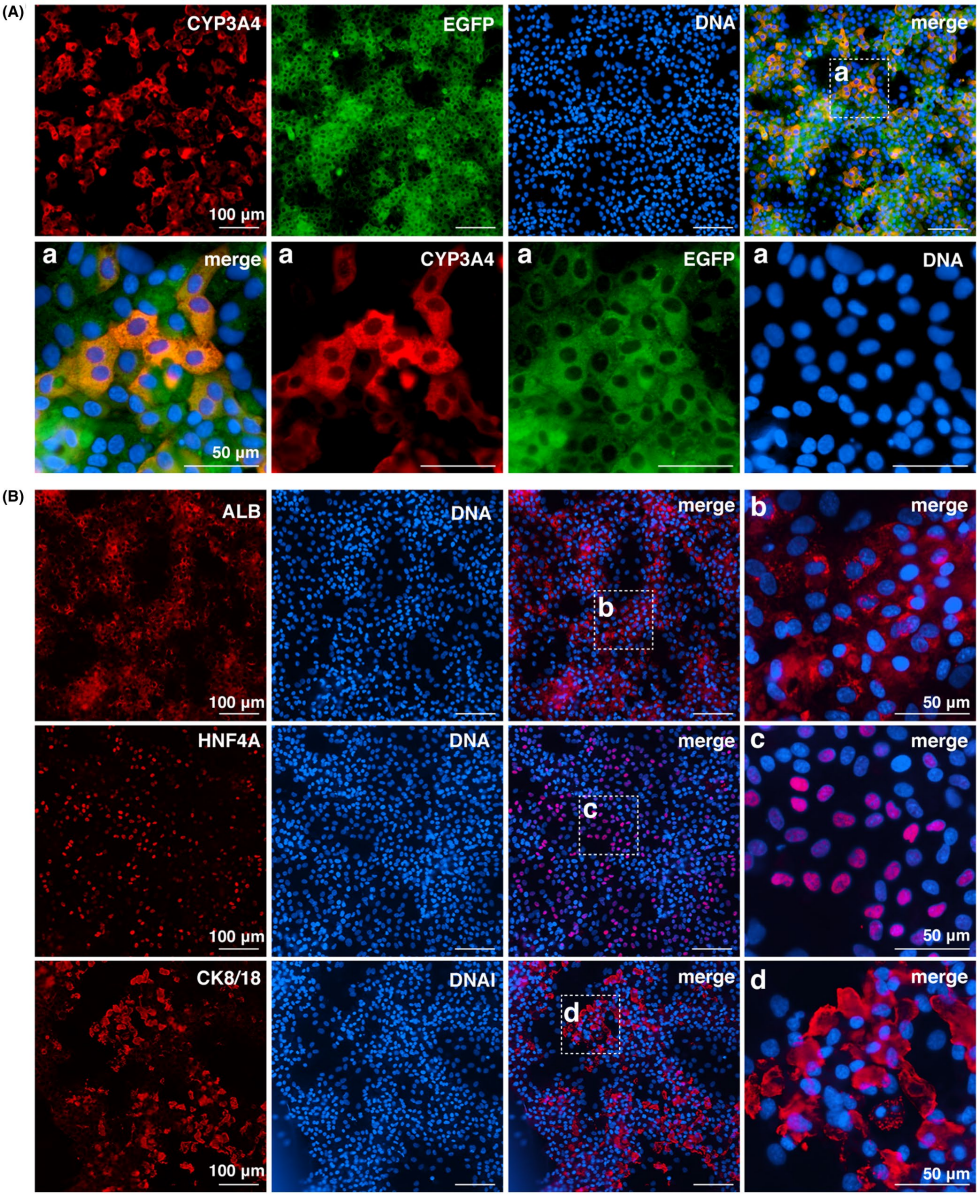
Protein expression for general hepatocyte‐specific markers in D10 HepaRG cells cultured on the O plate. A, CYP3A4 and EGFP; B, HNF4A, ALB, and CK8/18. a–d, An enlarged view of the center of the well is shown in A and B

To determine whether D10 cells that were cultured on the O plate reliably differentiated into HLCs, the cells were subjected to an ICC analysis for representative markers of mature human PHHs. Most of D10 cells were positive for CYP3A4, HNF4A, CK8/18, and ALB (Figure 3).

### 3.3 Effect of plate type on the fluorometric *CYP3A4* transcriptional induction test

**FIGURE 4:**
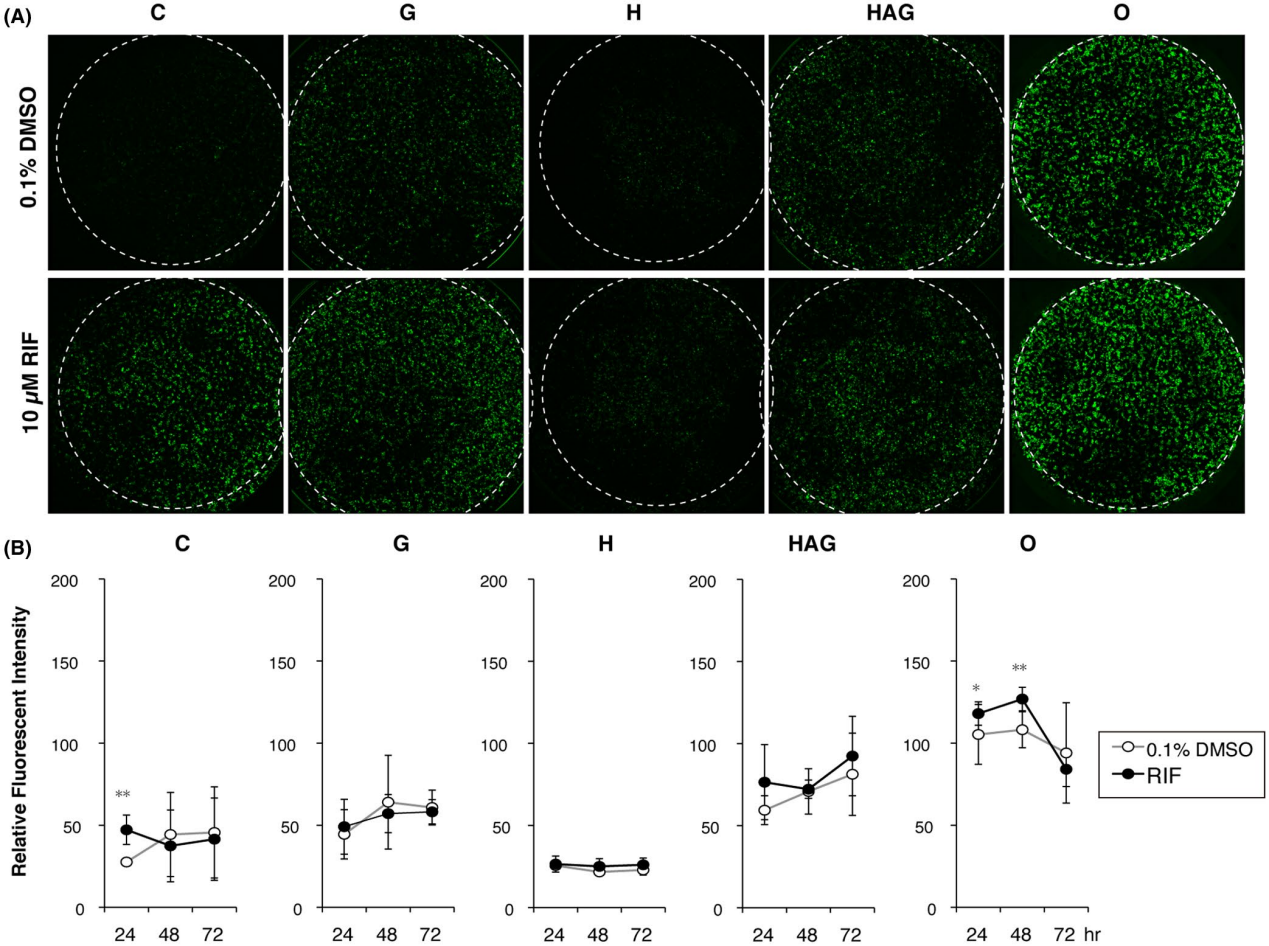
Effect of culture plate type on fluorometric *CYP3A4* transcriptional induction tests. A, Images of EGFP‐positive cells were captured on D10 after culturing in medium containing 0.1% DMSO (upper, untreated control) or 10 µmol L^−1^ rifampicin (RIF) (bottom, treated sample) for 48 hours. B, Semi‐quantitative fluorometric evaluation of EGFP‐positive hepatocyte‐like cells (HLCs). Each value was normalized to the mean value obtained in D9 cells cultured in O plates in the 1.7% DMSO medium as 100 (n = 3). Black circles, cells cultured in medium containing 10 µmol L^−1^ RIF; open circles, cells cultured in the 0.1% DMSO medium during the last 3 days of the cell culture period

After adding RIF or 0.1% DMSO, fluorescence images were captured at 24, 48, and 72 hours (Figure 4A). Image J was used to calculate total fluorescence per area at each time point in five plates. Significant transcriptional induction was only detected in cells cultured on C plates at 24 hours and on O plates at 24 and 48 hours. Transcription induction rates were 1.7‐, 1.1‐, and 1.2‐fold higher than those in the 0.1% DMSO control, respectively (Figure 4B). These results indicated that only HLCs cultured on C plates provided reliable results in the fluorometric *CYP3A4* induction test.

### 3.4 Effect of culture plate type on *CYP3A4* mRNA expression

**FIGURE 5:**
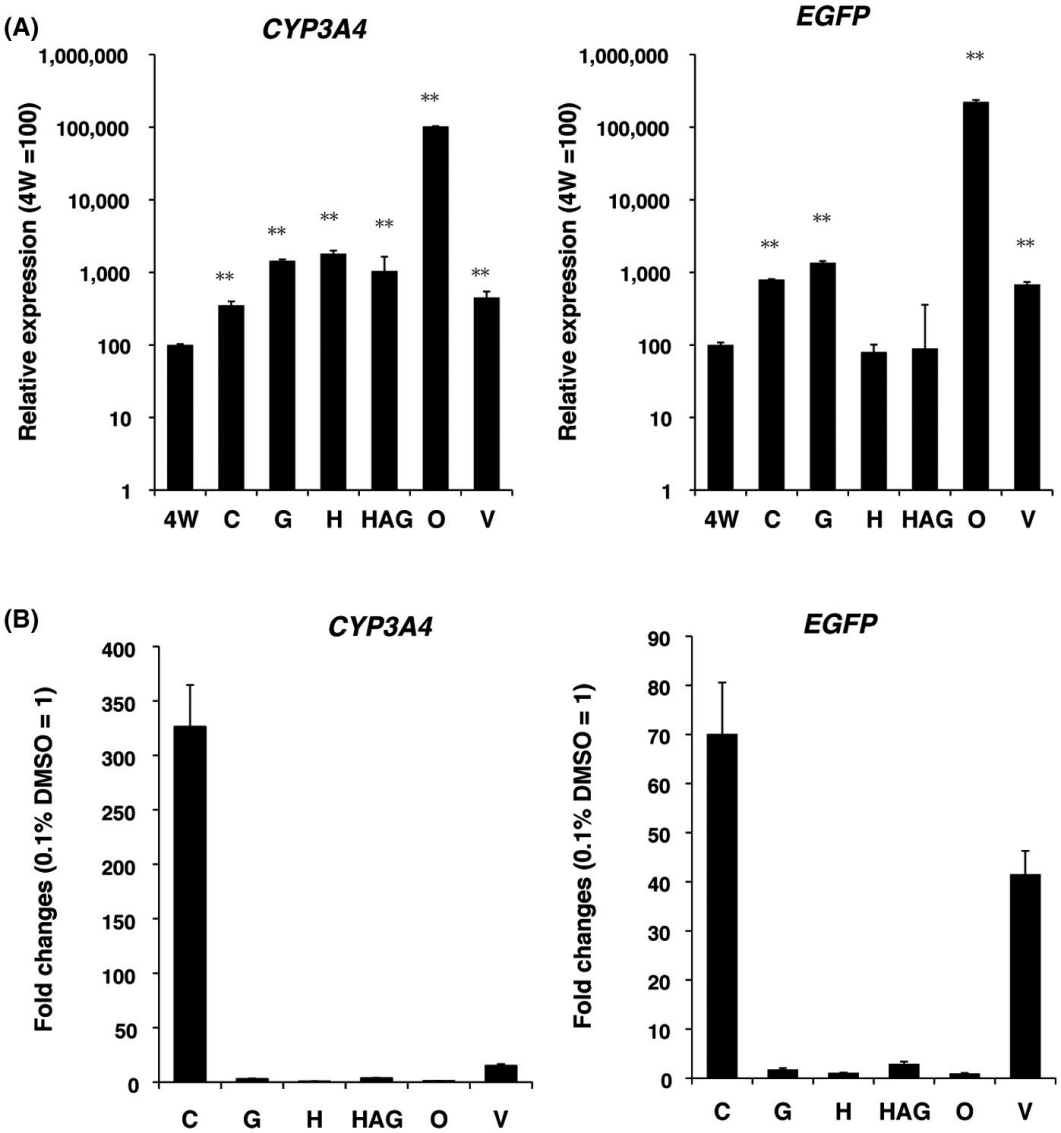
Effect of plate type on *CYP3A4* mRNA transcription assessed by RT‐qPCR. A, Enhanced expression of *CYP3A4* and *EGFP* mRNA evaluated by RT‐qPCR (n = 3, triplicate measurements). B, *CYP3A4* and *EGFP* transcriptional induction rates estimated according to mRNA levels. Graphs show the mean ± SD of the fold change with the 0.1% DMSO control considered as 1

RNA expression levels of endogenous *CYP3A4* and exogenous *EGFP* in D10 cells cultured on five 96‐well plates and one 24‐well V plate were compared with those in the original 4‐W HLCs. Total RNA was collected from the cells used for cell imaging. *CYP3A4* mRNA levels were significantly increased in all plates, including C plates (*P* < .001) (Figure 5A, left). *EGFP* expression was significantly increased in most of the plates except in H and HAG plates (Figure 5A, right). These results indicated that all plates used in the study upregulated *CYP3A4* mRNA expression in HLCs during 10 days of cell culture.

Next, we calculated the induction rate of *CYP3A4* on the basis of mRNA expression levels. *CYP3A4* and *EGFP* mRNA levels increased by more than 300‐ and 70‐fold, respectively, in RIF‐treated cells cultured on C plates compared with the 0.1% DMSO‐treated controls (Figure 5B). By contrast, *CYP3A4* mRNA induction rates were 3.0, 3.6, 1.1, and 15.0 in G, HAG, O, and V plates, respectively. No induction occurred in H plates. The results of RT‐qPCR and fluorometric analyses showed that *CYP3A4* transcription levels were already highly upregulated in HLCs cultured in the medium with 1.7% DMSO on O plates; therefore, it is possible that transcription could not increase further in HLCs in response to activation of nuclear receptors by RIF.

### 3.5 Effect of plate type on the metabolic activity of CYP3A4

**FIGURE 6:**
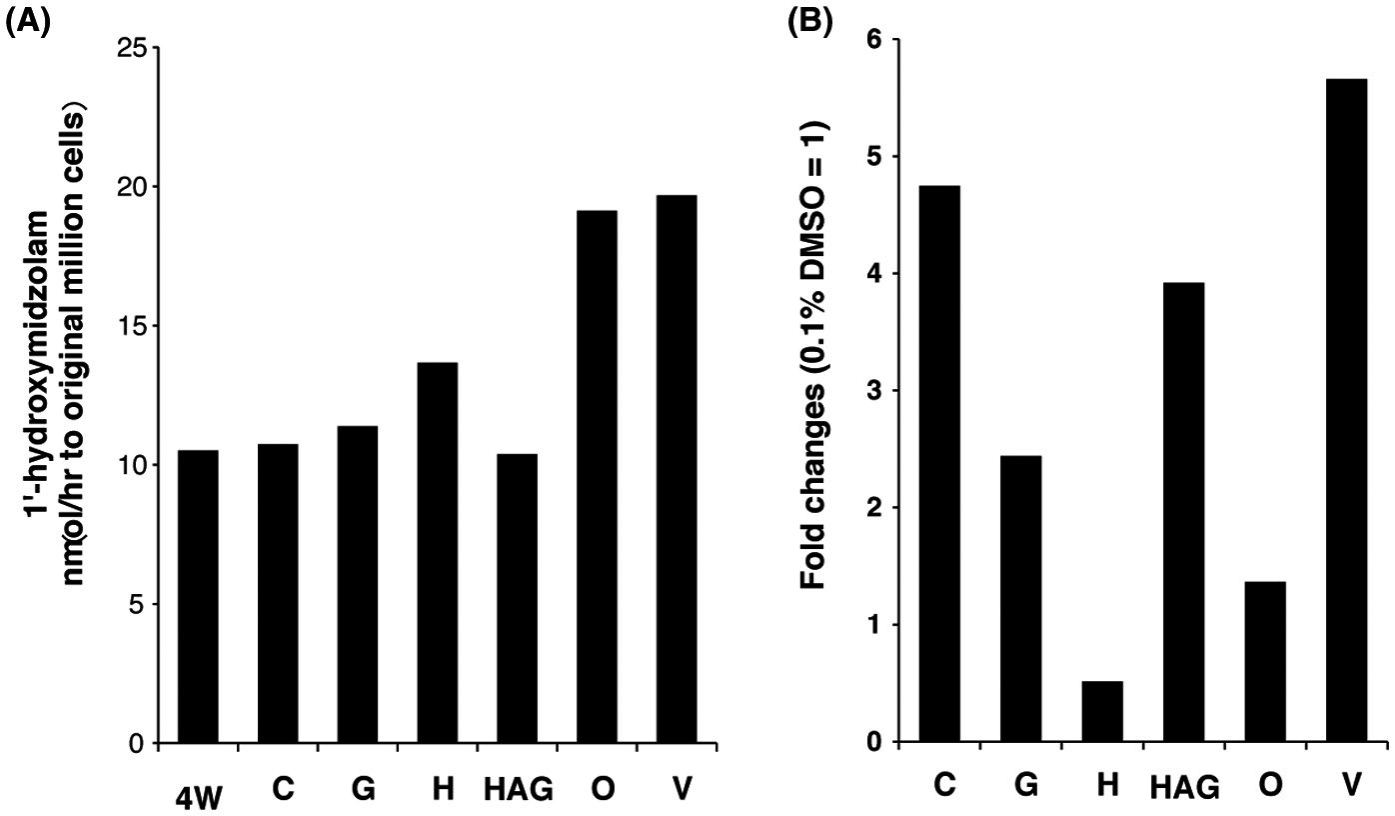
Effect of plate type on the metabolic activity of CYP3A4 assessed by LC‐MS/MS. A, Comparison of the amounts of the CYP3A4‐mediated metabolite midazolam (mixture from four wells). B, Induction rate of *CYP3A4* transcription estimated according to the amount of CYP3A4 metabolite after treatment with 10 μmol L^−1^ RIF for 48 hours

Midazolam was added to the medium of the six plate types to determine which plate would provide high‐quality HLCs that had CYP3A4 enzyme activity. The CYP3A4 metabolite 1'‐hydroxymidazolam, which is naturally excreted into the culture medium, was analyzed by LC‐MS/MS. The amount of metabolite excreted from HLCs cultured on O plates was twofold higher than that derived from the original 4‐W HLCs (Figure 6A). In addition, CYP3A4 activity in HLCs cultured in O plates was as high as that in HLCs cultured in 24‐well V plates under the 3D culture condition (Figure 6A).

The responsiveness of HLCs to RIF was evaluated by measuring the amount of 1'‐hydroxymidazolam. The induction rates were 5.7‐, 4.8‐, 3.9‐, 2.4‐, 1.4‐, and 0.5‐fold higher in HLCs cultured in V, C, HAG, G, O, and H plates, respectively (Figure 6B). Measurement of green fluorescence and metabolite amounts indicated a correlation between induction rates (R = 0.87, *P* = .05). The results of the screening to exclude unfavorable compounds regarding *CYP3A4* transcriptional induction potency indicated that culture of HLCs in glass‐bottom plates such as C plates is sufficient for the fluorometric *CYP3A4* induction test.

Additionally, 1.7%‐2% DMSO induces HepaRG cell differentiation and also *CYP3A4* expression. Thus, *CYP3A4* mRNA expression has already been induced in HepaRG HLCs in the 1.7% DMSO medium before it was applied for the induction test. This may be the reason why mature HLCs responded poorly to classical PXR activators. Thus, HLCs are usually cultured in a DMSO‐depleted medium to restore the lost *CYP3A4* inducibility before applying HLCs to induction studies. However, DMSO removal causes HLC dedifferentiation. Therefore, some of the compounds that enhance *CYP3A4* expression and EGFP fluorescence are possibly true PXR activators and/or maturation‐inducing compounds. Further functional details regarding candidate compounds should be precisely analyzed after the convenient fluorometric HCS‐based assay.

### 3.6 Optimization of 2D culture conditions for the formation of mini‐liver‐like structures containing HLCs and biliary epithelium on O plates

**FIGURE 7:**
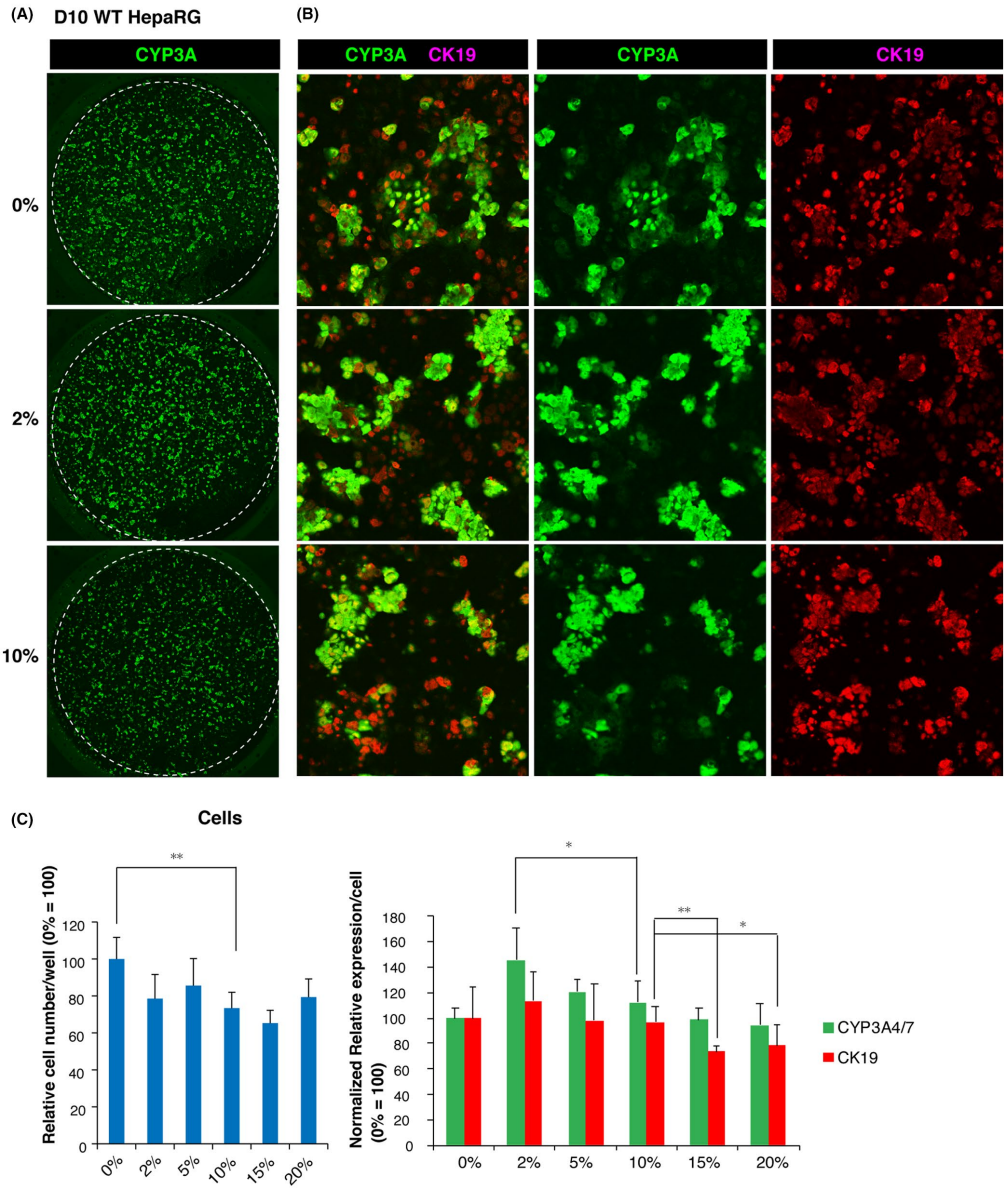
Optimized 2D culture system for the formation of hepatocyte‐like cells (HLCs) from HepaRG cells and biliary epithelium on O plates coated with 2% Cellmatrix Type I‐A. A, Images of CYP3A4‐positive cells (green) and CK19‐positive cells (red) generated by confocal microscopy (upper). An enlarged view of the center of the well is shown in B. C, Semi‐quantitative fluorometric evaluation of HLCs and the biliary epithelium according to the ICC signal intensity for CYP3A4 and CK19, respectively. Cell growth under the indicated conditions was estimated based on the fluorescence intensity of DNA staining with Hoechst 33 258 (cells). Normalized relative values were calculated according to the average value obtained from D10 cells cultured on noncoated O plates taken as 100 (n = 4)

To optimize the cell culture conditions of HepaRG‐derived HLCs, O plates were collagen‐coated with 0%, 2%, 5%, 10%, 15%, or 20% Cellmatrix Type I‐A (n = 4). In this experiment, D10 HLCs from WT HepaRG cells were subjected to ICC analysis using anti‐human CYP3A4 and human anti‐human CK19 antibodies, which are representative markers of mature HLCs and bile duct epithelium, respectively. ICC images were acquired using a confocal microscope (Figure 7A,B), and the mean relative fluorescence intensity normalized by DNA staining intensity for CYP3A4 and CK19 was calculated to evaluate the effects of collagen on HLC maturation (Figure 7C). In addition, cell growth, evaluated by the relative intensities of DNA staining, was 1.4 times higher in untreated plates than in 10% collagen‐coated wells. However, CYP3A4 and CK19 protein expression levels in uncoated plates were equivalent to those in 10% collagen‐coated plates. The maximum values for CYP3A4 and CK19 were obtained in 2% collagen‐coated O plates, which were approximately 1.3‐fold higher than those obtained in 10% collagen‐coated wells (n = 4, *P* < .01). These results indicated that O plates coated with 2% Cellmatrix Type I‐A were the most effective at promoting the maturation of HepaRG cells.

## 4 DISCUSSION

CYP3A4G/7R HepaRG is a transgenic cell line that was previously established in our laboratory. The use of transgenic HLCs allows the evaluation of relative CYP3A4 enzyme activity by measuring green fluorescence intensity in cell culture, thus avoiding cost‐ and time‐consuming analyses. In this study, we used this screening system and found that the 96‐well Optical CVG plates referred to as O plates with 2% collagen coating maximized CYP3A4 activity in CYP3A4G/7R and WT HepaRG‐derived HLCs during additional 10‐day cultures. Therefore, the combination of Optical CVG plates and HepaRG‐derived HLCs allows evaluation of different aspects of many candidate compounds simultaneously.

HepaRG is the only cell line capable of providing unlimited numbers of HLCs expressing CYP3A4 at high levels. Cryopreserved 4‐W HepaRG‐derived HLCs are commercially available from Biopredic International and can replace PHHs depending on the accuracy required for testing. However, HLCs show partial loss of cell maturity after defrosting and reentry into mitosis. Thus, cell culture for recovering maturity is necessary on assay plates. In this study, we found that high‐density cell culture of freshly prepared 4‐W HLCs significantly increased the number of EGFP‐positive cells even under 2D cell culture conditions. Although the Preset VECELL® referred to as a V plate cannot be used for HCS‐based evaluation, it significantly enhanced CYP3A4 activity. Thus, V plates are suitable for the preparation of large amounts of functional HLCs from HepaRG cells, as previously shown in PHH cultures.^19^

The development of HCS system using 3D‐cultured hepatocytes remains problematic, as suspended spheroids are easy to lose in long cell culture and it is also difficult to capture representative cellular images for each HLC spheroid showing an average response to a given drug. Moreover, effective gas supply to the center of cell aggregates is necessary to avoid apoptosis under 3D cell culture conditions. To resolve this issue, G, H, and HAG (VECELL®) plates were developed with a PDMS or fibrous flat bottom. In cell transplantation therapy, HLC spheroids are formed in coculture with mesenchymal stem cells, and vascular endothelial cells are used to enhance the function of HLCs and prolong the life of transplanted cells.^20^ Therefore, the results showing that HLCs cultured on Optical CVG plates expressed CYP3A4 at high levels under 2D culture conditions are a new discovery deviating from the existing concept. In addition, we found that cell culture conditions that upregulate *CYP3A4* expression to nearly the highest levels render HLCs insensitive to nuclear receptor‐mediated transcriptional activation, leading to false‐negative results. This suggests that, for fluorometric *CYP3A4* induction tests, the use of standard multiwell plates with a standard bio‐coat bottom might be sufficient.

Further investigation is necessary to determine whether this phenomenon is HepaRG specific or if it occurs in human PHHs. In addition to HepaRG, attempts have been made to produce functional adult‐type HLCs from human‐induced pluripotent stem cells (iPSCs), although mature HLCs remain to be produced from human iPSCs.^21^, ^22^ Therefore, the establishment of an in vitro system capable of promoting HLC maturation would be favorable. The 2D culture system presented here may also be effective for the maturation of human iPSC‐derived HLCs.

The use of CYP3A4G/7R HepaRG‐derived HLCs cultured in the 96‐well glass‐bottom plates selected in this study enables semi‐quantitative evaluations of compounds based not only on fluorescence, but also on high levels of enzyme activity. This system would enable additional analyses such as cell death caused by hepatotoxicity and metabolite evaluation.

## DISCLOSURE

The authors declare no conflict of interest.

## AUTHOR CONTRIBUTIONS

Masako Tada, the corresponding author, designed the research, involved in final proofreading before submission, collected the data, and contributed to data analysis. Keiko Ooeda, Mao Yamashita, and Shota Okuyama conducted the experiments, performed data analysis, and contributed to the writing of the manuscript. Musashi Kubiura‐Ichimaru, Akari Mine, Saori Tsuji, and Takafumi Ueyama conducted experiments and performed data analysis. Fumihiko Kawamura contributed new reagents or analytic tools.

## ETHICAL STATEMENT

The authors declare that this study was performed in accordance with the research policy of Toho University.

### OPEN RESEARCH BADGE

This article has earned an Open Data and Open material badges for making publicly available the digitally‐shareable data necessary to reproduce the reported results. All data and materials are available in the article.

## Data Availability

All data discussed in this publication is included in this manuscript. Further information and requests for data and reagents should be requested to the corresponding author, Masako Tada. Please contact masako.tada@sci.toho‐u.ac.jp.
